# The Incidence and Mortality of Cervical Cancer in Ningbo during 2006–2014, China

**Published:** 2017-10

**Authors:** Hui LI, Donghui DUAN, Jiaying XU, Qinghai GONG, Yong WANG, Wei JI, Lingbin DU, Liyuan HAN, Guozhang XU

**Affiliations:** 1. Institute of Non-Communicable Diseases Control and Prevention, Ningbo Municipal Center for Disease Control and Prevention, Ningbo, China; 2. School of Medicine, Zhejiang Provincial Key Laboratory of Pathophysiology, Ningbo University, Ningbo, China; 3. Dept. of Epidemiology, School of Public Health and Tropical Medicine, Tulane University, New Orleans, USA; 4. Zhejiang Cancer Center, Hangzhou, China

**Keywords:** Cervical cancer, Incidence, Mortality, China

## Abstract

**Background:**

The purpose of this study was to investigate the incidence and mortality rates of cervical cancer during 2006–2014 in Ningbo, China.

**Methods:**

A retrospective study involved 3418 newly diagnosed cervical cancer cases and 854 death cases were performed. All cases were registered in Cancer Registry Center of Ningbo Centers for Disease Control and Prevention. Results were expressed as standardized age-specific cancer incidence/mortality rates with confidence intervals. All *P-*values presented were two-sided and the statistical significance was set at *P*<0.05.

**Results:**

The crude incidence rate was 34.35 per 100000 in females aged 50–54 years. Females aged 80–84 years had the highest crude mortality rates, which were 12.91 per 100000. The average age-standardized incidence and mortality rates by Chinese Standard Population were 6.29 and 1.49 per 100000, respectively. The average age-standardized incidence and mortality rates by World Standard Population were 8.02 and 1.91 per 100000, respectively. The incidence trend graph showed that annual percent change (APC) increased rapidly by 30.2% (*P*<0.01) during 2006–2014, while the mortality trend graph indicated a rapid increase in mortality annually by 8.8% (*P*<0.01) during 2006–2014.

**Conclusion:**

We observed an increased trend for both incidence and mortality rates of cervical cancer in Ningbo during 2006 to 2014, which indicated the urgent need for free regular screening for high-risk populations by the government.

## Introduction

Cervical cancer is the third most frequently diagnosed cancer in females, and the fourth leading cause of cancer deaths globally (1). According to the research of the National Cancer Center, there were nearly 75500 newly diagnosed cervical cancer patients and 34000 deaths every year in China (2).

In developed countries, due to the widespread and systematic implementation of Papanicolaou (Pap) testing, the overall incidence rate of cervical cancer has been declining (3, 4). Unlike those developed countries, China experienced an increased upward trend of the incidence and mortality rates of cervical cancer during the past two decades (5), especially among the younger females (6–8). Cervical cancer has been the second leading cause of death in females (9). WHO suggested that high coverage of 80% of the population at risk of cervical cancer was an important factor for a successful screening program (10). However, only around 19% of eligible rural females coverage in the national rural screening program in China (11).

In 2008, the incidence of cervical cancer was 11.87 per 100000 in China (12), while the incidence was 13.89 per 100000 in Zhejiang Province (13). Ningbo is an economic center of Zhejiang Province, which is also a coastal city with population over than 7 million, with a high level of economic development comparing to the general situation of China (GDP per capita in 2014: 16,112$ vs 7065$) (14, 15). This is a high-incidence region for cervical cancer in China. The adjusted incidence rate of cervical cancer was 20.11 per 100000 in 2010 (16). To date, there are few data about the specific incidence and mortality rates of cervical cancer in Ningbo. Therefore, our investigation focused on the incidence and mortality rates of cervical cancer during 2006 to 2014 in Ningbo.

We aimed to provide evidence for the implementation of early screening and effective intervention strategies for the local government.

## Materials and Methods

### Study design

The patients of this study were limited to Ningbo residents, diagnosed with cervical cancer and registered in Ningbo Cancer Registry Center during Jan 1, 2006, to Dec 31, 2014. The source data (including the information of cases and population estimates) were obtained from Ningbo Centers for Disease Control and Prevention. Those who were non-Ningbo residents and repeatedly registered cases were excluded. The incidence and mortality data were divided into the following age groups: below 1, 1–4, 5–9, 10–14, 15–19, 20–24, 25–29, 30–34, 35–39, 40–44, 45–49, 50–54, 55–59, 60–64, 65–69, 70–74, 75–79, 80–84, and 85 years or older. All cases were coded according to the International Classification of Diseases, 10th Revision (ICD-10) (17).

All patients gave their written informed consent, and this study was conducted in accordance with the principles of the Declaration of Helsinki.

### Statistics

The numbers of new cases and deaths were estimated using the 5-years age-specific cancer incidence/mortality rates along with the relevant populations in each group. The crude, standard, and age-specific incidence and mortality rates were calculated by the direct method using Excel 2007 (Microsoft Office 2007, Microsoft Corporation, USA). The trends in incidence and mortality were analyzed by Joinpoint Regression Program 4.3.1.0 produced by National Cancer Institute (http://www.surveillance.cancer.gov/joinpoint/). The annual percent change (APC) was calculated using the Joinpoint Regression model. The rest data were described and analyzed by Empower(R) (www.empowerstats.com, X&Y solutions, inc. BostonMA) and R (http://www.R-project.org). All rates were expressed in per 100000 population and were directly age-adjusted to the Chinese population in 1982 and World Segi’s population, respectively. Statistical significance was set at a *P*≤0.05.

## Results

The demographic data is shown in Table 1. There were 3418 newly diagnosed cervical cancer cases and 854 death cases, with mean ages being 55.37±11.85 and 59.60±14.12 years, respectively. The age distribution of all cervical cancer incidence-cases was 1.9% aged less than 34 years, 14.8% aged 35–44 years, 38.9% aged 45–54 years, 25.9% aged 55–64 years and 18.5% aged larger than 65 years (Table 1). The highest incidence and death cases were in the age group of 35–69 years. The ratio of mortality to incidence was 24.99% in this study.

**Table 1:** Baseline characteristics of the cervical cancer cases and deaths

***Variables***	***N (%)***	***Age (Mean±SD)***
Incidence cases	3418	55.37±11.85
≤34	66(1.9%)	31.35±5.23
35–44	506(14.8%)	41.24±2.72
45–55	1329(38.9%)	50.03±2.81
55–65	885(25.9%)	59.78±2.70
≥65	632(18.5%)	74.23±7.78
Death cases	854	59.60±14.12
≤34	25(2.9%)	30.96±3.65
35–44	130(15.2%)	42.09±2.62
45–55	198(23.2%)	49.84±2.44
55–65	241(28.2%)	59.49±3.15
≥65	260(30.4%)	76.57±6.75
Marriage(only death cases)	854	59.60±14.12
Single	6(0.7%)	43.89±27.12
Married	560(65.6%)	55.61±12.61
Widowed	269(18.2%)	73.97±12.20
Divorced	19(15.5%)	46.89±8.37
Education(only death cases)	854	59.60±14.12
≤Middle school	611(71.5%)	62.90±13.25
High school	189(22.1%)	46.43±9.05
≥University	54(6.4%)	39.92±11.06

### Crude incidence rate (CIR) and mortality rate (CMR)

The crude rates of incidence and mortality were 11.82 and 2.95 per 100000 during 2006 to 2014, respectively. Table 2 shows the crude incidence and mortality rates of cervical cancer among different age groups. The crude incidence rate was 19.30 per 100000 in 2013, which was the highest between the years 2006 to 2014. However, the crude mortality rate was the highest in 2014 with a rate of 4.50 per 100000. Patients aged 50–54 years had the highest crude incidence rate among all age groups, which were 34.35 per 100000. Among patients in the age group 80–84 years, the crude mortality rate was the highest.

**Table 2:** The crude incidence and mortality rates of cervical cancer in Ningbo for all years

***Age group (yr)***	***CIR***	***CMR***
0-	0.00	0.00
1-	0.14	0.00
5-	0.00	0.00
10-	0.00	0.00
15-	0.07	0.07
20-	0.24	0.00
25-	0.32	0.21
30-	2.33	0.88
35-	7.22	1.51
40-	12.97	2.79
45-	23.95	5.10
50-	34.35	4.80
55-	23.10	5.86
60-	25.61	6.43
65-	25.40	6.33
70-	20.45	6.82
75-	11.75	9.99
80-	18.54	12.91
85-	24.61	9.00

CIR=Crude incidence rate, CMR=Crude mortality rate

### Age-standardized incidence and mortality rates

The age-standardized incidence and mortality rates were presented in Table 3. The average age-standardized incidence rate by Chinese Standard Population (ASIRC) was 6.29 per 100000.

**Table 3:** The age-standard incidence and mortality rates during 2006–2014 in Ningbo

***Year***	***CIR***	***CMR***	***ASIRC***	***ASMRC***	***ASIRW***	***ASMRW***
2006	2.46	2.11	1.12	1.00	1.48	2.37
2007	3.46	2.70	1.61	1.36	2.15	3.12
2008	5.64	2.70	2.67	1.31	3.56	3.23
2009	11.23	3.16	5.70	1.47	7.54	3.41
2010	15.57	3.80	7.41	1.87	9.61	4.35
2011	15.90	3.11	7.83	1.50	9.95	3.90
2012	18.56	3.29	9.13	1.60	11.54	3.83
2013	19.30	4.20	9.55	1.94	11.94	5.03
2014	18.38	4.50	8.93	1.95	11.13	5.24

CIR=Crude incidence rate, CMR=Crude mortality rate, ASIRC=Age-standard incidence rate by Chinese Standard Population, ASMRC=Age-standard mortality rate by Chinese Standard Population, ASIRW=Age-standard incidence rate by World Standard Population, ASMRW=Age-standard mortality rate by World Standard Population

The average age-standardized mortality rate by Chinese Standard Population (ASMRC) and World Standard Population (ASMRW) were 1.49 and 1.82 per 100000, respectively.

Among patients aged 0–74 years, the cumulative incidence rate was 8.81%. The truncated rate for 35–64 years age group was 19.99 per 100000. The average age-standardized mortality rate by Chinese Standard Population (ASMRC) was 1.49 per 100000. The average age-standard mortality rate by World Standard Population (ASMRW) was 1.82 per 100000. The cumulative mortality rate for 0–74 years age group was 2.04%. The truncated rate for 35–64 years age group was 3.43 per 100000. After age standardized of the data, both ASIRC and ASIRW had their peak values in 2013, whereas ASMRC and ASMRW had the highest ratio in 2014.

### Incidence and mortality trends from 2006 to 2014

**Fig. 1:**
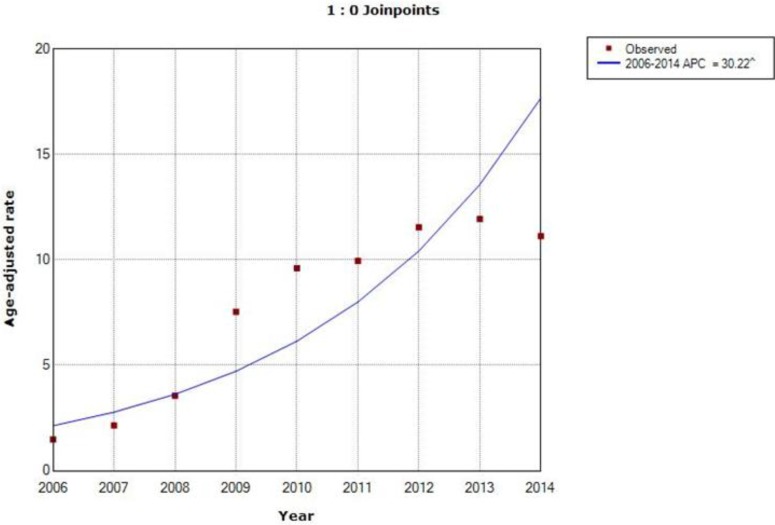
Incidence trend graph of cervical cancer in Ningbo during 2006–2014 APC=Annual percent change

**Fig. 2:**
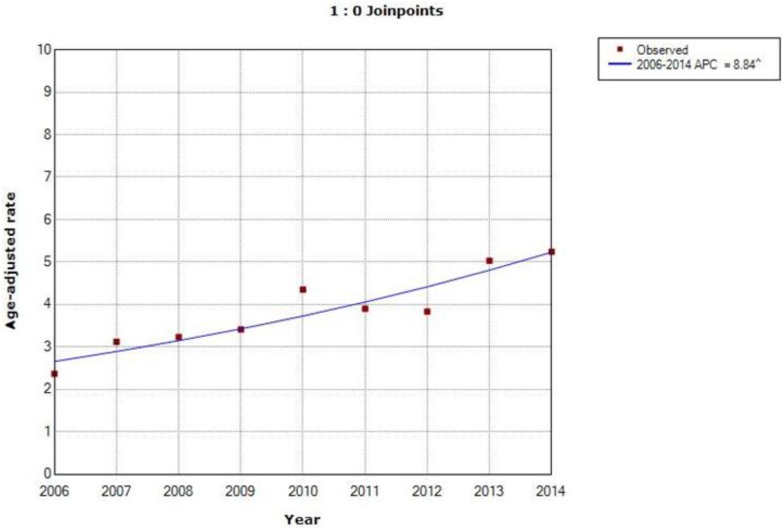
Mortality trend graph of cervical cancer in Ningbo during 2006–2014 APC= Annual percent change.

The ASIRW of cervical cancer were 1.48 and 11.13 per 100000 in 2006 and 2014, respectively. Overall, an increased trend in incidence was observed. The incidence trend graph Fig. 1 showed the APC increased rapidly by 30.2% (*P*<0.01) during 2006–2014. The lower and upper confidence interval (CI) was presented in Table 4. The ASMRW of cervical cancer were 2.37 and 5.24 per 100000 in 2006 and 2014, respectively. The mortality trend graph Fig. 2 suggested a rapid increase in mortality annually by 8.8% (*P*<0.01) during 2006–2014. And the lower and upper CI was presented in Table 4.

**Table 4:** Joinpoint incidence and mortality trend analysis of cervical cancer

***Type***	***Segment***	***Lower Endpoint***	***Upper Endpoint***	***APC***	***Lower CI***	***Upper CI***	***Test Statistic (t)***	***P***
Incidence	1	2006	2014	30.2	16.7	45.4	5.7	<0.001
Mortality	1	2006	2014	8.8	5.6	12.1	6.7	<0.001

CI=Confidence interval; APC=Annual percent change.

## Discussion

We observed a persistent increased trend of cervical cancer incidence and mortality rates during 2006–2014 in Ningbo. Consistent with our study, the CIR in China was 3.06 per 100000 in 1990, but it rocketed to 11.87 per 100,000 in 2008 rapidly(12). While the CMR was slightly increased from 2.19 per 100000 in 1990 to 3.20 per 100000 in 2008 in China (18). Interestingly, cervical cancer appeared to be increased among younger females. The median age of incidence cases was 67 years in 1993, but it decreased to 45 years in 2008 (6). Besides, the peak of the age-specific incidence curve decreased to 40 years in 2005–2008, which was around 25–29 years earlier than seen in 1993–1996.6. The prevalence of cervical cancer was increased in young females (18). Similarly, the highest proportion of cervical cancer incidence cases was among the 45–49 age group in Zhejiang Province (13). Although the peak of incidence has patients aged 50–54 years in our study, it was much younger than 20 years earlierr (6).

In our study, the mean age of incidence cases was 55.37±11.85 years and the highest incident rate was among females aged 50–54. Contrary to our study, the incidence rate in national urban areas was the highest in the age group of 40–44, while the national rural incidence rate was the highest in the 55–59 age group according to National Cancer Statistic Center (19). This discrepancy might be due to the different environmental and lifestyle factors. In recent years, more and more oil depots were put into use in Ningbo, which resulted in a dramatic increase of petroleum and other marine pollutants (20). Furthermore, these pollutants have carcinogenicity and threaten the safety of seafood. Ningbo is a coastal city and the local residents consume variety of seafood daily. Undoubtedly, a positive relationship between high incidence and high mortality rates was observed. Cervical cancer was influenced by the extrinsic, environmental, and lifestyle factors (21). Among those factors, human papillomavirus (HPV) is considered to be the primary cause(22–24). The female who was positive for HPV DNA had a risk of developing cervical cancer 15–50 times higher than those without HPV DNA(25). Fortunately, effective vaccines are now available to prevent the infection of this disease due to HPV (26, 27).

Along with rapid economic development and urbanization, the medical and health care in China has been improved remarkably in the past three decades. A continuous decreasing trend of cervical cancer was observed incidence and mortality rates in China from 1970 to 2004 (8), the same trend was also observed in some Asian countries (28–31). However, in our study, we witnessed an increased trend for incidence rate. The incidence trend graph showing the incidence increased annually by 30.2% (*P*<0.01) during 2006–2014. The increase might be due to the examination of gynecological diseases performed in 2007 in Ningbo and the screening program for cervical cancer performed by the China government in 2009. These examinations aimed to identify and treat gynecological diseases at early stages and to improve females’ quality of life. Besides, residents in Ningbo had low levels of cognition of risk factors and early screening of cervical cancer. Only 28.10% of all informants knew HPV infection as a high-risk factor for cervical cancer (32). Approximately 50% of informants were willing to afford the cost of 14.81 dollars for the screening, however, the cost of Thin-Cytologic Test (TCT) and HPV-DNA Test was nearly 300 Yuan (32). Free charges of cervical cancer screening for the rural females aged 35–59 is necessary (33).

Notably, in 2009, the age-standard incidence rate was 7.54 per 100000, which was over two times higher than the incidence rate of last year. The screening program for cervical cancer performed by the China government in 2009 contributed to the increased detection of incidence rate. Age, occupation, cultural level, and economic conditions had great influence on the cognition level of the population (32). In our study, 71.5% of death cases had low educational level. Furthermore, population including unemployed people, workers, and farmers were at higher risk (6). And those people tend to have lower educational level, poorer income, poor health condition, and lack awareness to get regular early cervical cancer screening (6). The Department of Health and Family Planning Commission should focus on the high-risk populations.

In developed countries, most cervical cancers can be avoided or diagnosed early through screening and treatment (34). Owing to the appropriate screening and follow-up treatment, the documented cervical cancer incidence, and mortality rates had been declined significantly (34, 35). According to the WHO, 80% of 100% coverage of the target population with Pap screening and an organized network for adequate diagnosis and treatment would allow a 60% to 90% reduction in cervical cancer. Thus, early screening, diagnosis, and effective treatments should be implemented comprehensively.

The strengths of our study were as follows: 1) this is the first study to investigate the incidence and mortality rates of cervical cancer in Ningbo. 2) This study covered all of the newly diagnosed cervical cancer and death cases, which could represent the epidemiology of cervical cancer in Ningbo. 3) The nature of this prospective study enabled us to obtain a less biased data. 4) Our findings provided the direct evidence of cervical cancer control and prevention for the Department of Health and Family Planning Commission in Ningbo.

However, our study had some limitations. 1) Some baseline information was missed in our data. 2) For the newly diagnosed patients, biochemical measurements were absent. 3) Cervical cancer was influenced by many factors, including Human Papillomavirus infection, sexual behavior at an early age, multiple male sexual partners and smoking etc. (3). We were unable to explore the effect of confounding factors and potential interaction factors. Future functional research is needed to elaborate the mechanisms of the interactions among multiple risk factors.

## Conclusion

Effective cervical cancer control is a pressing issue in Ningbo. Early detection, diagnosis, and treatments for cervical cancer should be applied. If the screening program is performed and aimed at the high-risk populations, the public health resource could have played more favorable rules. Therefore, focusing on raising public awareness about cervical cancer screening and suitable treatments of cervical cancer is imperative.

## Ethical considerations

Ethical issues (Including plagiarism, informed consent, misconduct, data fabrication and/or falsification, double publication and/or submission, redundancy, etc.) have been completely observed by the authors.
